# Physiological hypoxia improves growth and functional differentiation of human intestinal epithelial organoids

**DOI:** 10.3389/fimmu.2023.1095812

**Published:** 2023-01-27

**Authors:** Gunnar Andreas Walaas, Shreya Gopalakrishnan, Ingunn Bakke, Helene Kolstad Skovdahl, Arnar Flatberg, Ann Elisabet Østvik, Arne Kristian Sandvik, Torunn Bruland

**Affiliations:** ^1^ Department of Clinical and Molecular Medicine (IKOM), NTNU - Norwegian University of Science and Technology, Trondheim, Norway; ^2^ Clinic of Laboratory Medicine, St. Olav’s University Hospital, Trondheim, Norway; ^3^ Centre of Molecular Inflammation Research (CEMIR), NTNU - Norwegian University of Science and Technology, Trondheim, Norway; ^4^ Central Administration, St. Olav’s University Hospital, Trondheim, Norway; ^5^ Department of Gastroenterology and Hepatology, Clinic of Medicine, St. Olav’s University Hospital, Trondheim, Norway

**Keywords:** oxygen, intestinal epithelial cells (IECs), differentiation, proliferation, inflammatory bowel disease, transcriptome, single-cell RNA-sequencing (scRNAseq), chemokines (cytokines)

## Abstract

**Background:**

The epithelium in the colonic mucosa is implicated in the pathophysiology of various diseases, including inflammatory bowel diseases and colorectal cancer. Intestinal epithelial organoids from the colon (colonoids) can be used for disease modeling and personalized drug screening. Colonoids are usually cultured at 18-21% oxygen without accounting for the physiological hypoxia in the colonic epithelium (3% to <1% oxygen). We hypothesize that recapitulating the *in vivo* physiological oxygen environment (i.e., physioxia) will enhance the translational value of colonoids as pre-clinical models. Here we evaluate whether human colonoids can be established and cultured in physioxia and compare growth, differentiation, and immunological responses at 2% and 20% oxygen.

**Methods:**

Growth from single cells to differentiated colonoids was monitored by brightfield images and evaluated with a linear mixed model. Cell composition was identified by immunofluorescence staining of cell markers and single-cell RNA-sequencing (scRNA-seq). Enrichment analysis was used to identify transcriptomic differences within cell populations. Pro-inflammatory stimuli induced chemokines and Neutrophil gelatinase-associated lipocalin (NGAL) release were analyzed by Multiplex profiling and ELISA. Direct response to a lower oxygen level was analyzed by enrichment analysis of bulk RNA sequencing data.

**Results:**

Colonoids established in a 2% oxygen environment acquired a significantly larger cell mass compared to a 20% oxygen environment. No differences in expression of cell markers for cells with proliferation potential (KI67 positive), goblet cells (MUC2 positive), absorptive cells (MUC2 negative, CK20 positive) and enteroendocrine cells (CGA positive) were found between colonoids cultured in 2% and 20% oxygen. However, the scRNA-seq analysis identified differences in the transcriptome within stem-, progenitor- and differentiated cell clusters. Both colonoids grown at 2% and 20% oxygen secreted CXCL2, CXCL5, CXCL10, CXCL12, CX3CL1 and CCL25, and NGAL upon TNF + poly(I:C) treatment, but there appeared to be a tendency towards lower pro-inflammatory response in 2% oxygen. Reducing the oxygen environment from 20% to 2% in differentiated colonoids altered the expression of genes related to differentiation, metabolism, mucus lining, and immune networks.

**Conclusions:**

Our results suggest that colonoids studies can and should be performed in physioxia when the resemblance to *in vivo* conditions is important.

## Introduction

The colon harbors an abundant microbial community adjacent to numerous immune cells, only separated by a mucous layer with a single-layered epithelium (1). The colonic epithelial cells are polarized, with an apical side facing the lumen and a basolateral side facing the lamina propria and orchestrate crosstalk between the microbial and immune cells communities (2). Continuously proliferating stem cells reside at the bottom of the crypts of Lieberkühn, giving rise to progenitor cells that migrate towards the lumen as they differentiate into postmitotic specialized epithelial cells (3, 4), including absorptive-, goblet-, enteroendocrine- (EEC) and tuft cells (5). Terminally differentiated epithelial cells undergo apoptosis and exfoliate into the lumen after two to five days, thus demanding a high turnover rate from the intestinal stem cells (6). Stem cell renewal and proliferation are stimulated by WNT signaling (4). Progenitor cells can commit to an absorptive or secretory lineage depending on the signaling molecules present. Central to the process is Notch signaling. Briefly, when the Notch molecule is present, the absorptive lineage dominates, and when Notch is suppressed, the secretory line is favored (7). Absorptive colonocytes are the fate of the absorptive lineage, while the secretory lineage differentiates into goblet-, EEC, or tuft cells. The differentiation of the secretory lineage also depends on other signaling molecules (4, 7).

The colonic epithelium is involved in the pathophysiology of inflammatory bowel disease (IBD), microscopic colitis, and colorectal cancer (8–10). Colorectal cancer is the third most prevalent cancer type globally, constituting 10.7% of all cancers diagnosed in 2020 (11). Thus, the colonic epithelium is central to cancer and inflammation research. The last decade has seen significant progress in gastrointestinal pathophysiology research with the introduction of 3D structured human intestinal organoid systems (12). Intestinal epithelial organoids (IEOs) mimic the epithelium’s architecture, cell composition, and signaling (12). IEOs from tissue-derived stem cells can recapitulate interindividual differences. Hence, they represent a tool in precision medicine for drug screening and can be used to discover how genetic and epigenetic variations influence pro-inflammatory responses (12–17). Culturing human IEOs from the colon (colonoids) *in vitro* has enabled researchers to study human colonic epithelial cell mechanisms directly, thus gaining new knowledge about colorectal cancer (18), IBD (19), and other diseases involving the intestinal epithelium (20). The advantages of human colonoids include ethical, economic, and applicability aspects (21). However, the strength of a model system lies in its ability to mimic *in vivo* conditions.

In the colonic epithelium, an oxygen gradient exists from the blood vessels in the submucosa (~3% oxygen), decreasing towards the luminal epithelial cells (< 1% oxygen) that are in juxtaposition to anaerobic bacteria (22). Thus, the colonic epithelium is adapted to thrive in a hypoxic environment (23). A central transcription factor for the epithelial adaptation to physiological hypoxia is Hypoxia-inducible factor-1 (HIF-1) (23). HIF-1 is a heterodimer formed by binding the oxygen-regulated HIF-1α and the continuously expressed HIF-1β. During low oxygen conditions, HIF-1α is stabilized, thus entering the nucleus, forming a heterodimer with HIF-1β. In the presence of high oxygen tensions, HIF-1α is continuously degraded (24). The interplay between the microbiome and colonic epithelium is partly responsible for stabilizing HIF-1 (25). The intestinal epithelium utilizes short-chained fatty acids (SCFA) produced by the microbiome for energy (26). Through β-oxidation of butyrate, local oxygen is depleted, and HIF-1α is stabilized (25, 27). HIF-1 is crucial for cell survival, metabolism, and other functions in low oxygen environments, including maintaining epithelial barrier integrity and antimicrobial functions (27). Stabilization of HIF-1α upregulates the expression of, e.g., thigh junction proteins (28), mucus-related genes (29), and anti-microbial proteins like defensins (30).

Preclinical studies have shown that stabilization of HIF-1α leads to improved intestinal barrier functions (31), and gut-targeted HIF-1α stabilizers like GB004 may be a promising therapeutic approach for ulcerative colitis patients (32). Recently, Kumar et al. (33) demonstrated that tumor cells from e.g. mouse colon and mammary tissue collected, processed, and propagated at physioxia (3% oxygen) displayed distinct differences in crucial signaling networks, including LGR5/WNT, YAP, and NRF2/KEAP1 and sensitivity to targeted therapies compared to tumors in ambient air (21% oxygen). The authors concluded that evaluating cancer cells under physioxia could more closely recapitulate their physiopathologic status in the *in vivo* microenvironment. Although IEO studies are generally performed at 18-21% oxygen, several have examined the benefits of growing other *ex vivo* models in physioxia (34–37). For example, kidney organoids cultured in physiological hypoxia (7% oxygen) instead of 21% oxygen showed enhanced sprouting and interconnectivity while maintaining renal cell types and their spatial organization (33). Primary human corneal endothelial cells can successfully be cultured at 2.5% oxygen resembling *in vivo* corneal aqueous environment containing 2.8% oxygen (37). Compared to room air (∼21% oxygen), corneal endothelial cells cultured at physiological hypoxia showed considerable differences in cell metabolism, viability, oxygen-consuming reactions, and glycolytic metabolism. Thus, the authors suggested that the culture of cells under conditions that most closely resemble their physiological environment would maintain the native phenotype and function and reduce external stressors.

Recently, we showed that short-time exposure (i.e. 40 hours) to physiological hypoxia (2% oxygen) did not alter viability and cell type expression in differentiated colonoids, but increased HIF-1α expression (38). Furthermore, differentiated colonoids cultured in physiological hypoxia for 40 hours expressed anti-inflammatory gene regulation traits upon TNF/IL17 stimulation. The present study aimed to evaluate whether physiological hypoxia should be a culture standard for human colonoids. First, we investigated if it was possible to culture human colonoids in a 2% oxygen environment throughout the culturing process. We assessed growth, colonoid cell composition, transcriptomic alterations, and response to pro-inflammatory stimulation. We then examined the direct effect of reducing the oxygen level on gene regulation in differentiated colonoids. Our findings support incorporating physioxia for human colonoid cultures.

## Materials and methods

Materials are listed in **Supplementary File 1** (SF1)

### Human colonoid cultures and experimental design

Colonoids were generated from human colonic biopsies as previously described (15, 38). For experiments, colonoids were dissociated into single cells, resuspended in ice-cold basement membrane matrix Matrigel GFR (Corning^®^, New York City, NY), plated on pre-warmed 24-well plates (8000-10000 cells in 50μl Matrigel per well), and 500μl of complete growth medium (CGM) was added to each well. The CGM is extensively described in previous publications (15, 38) and listed in **SF1**, sheet 2

**Figure 1:**
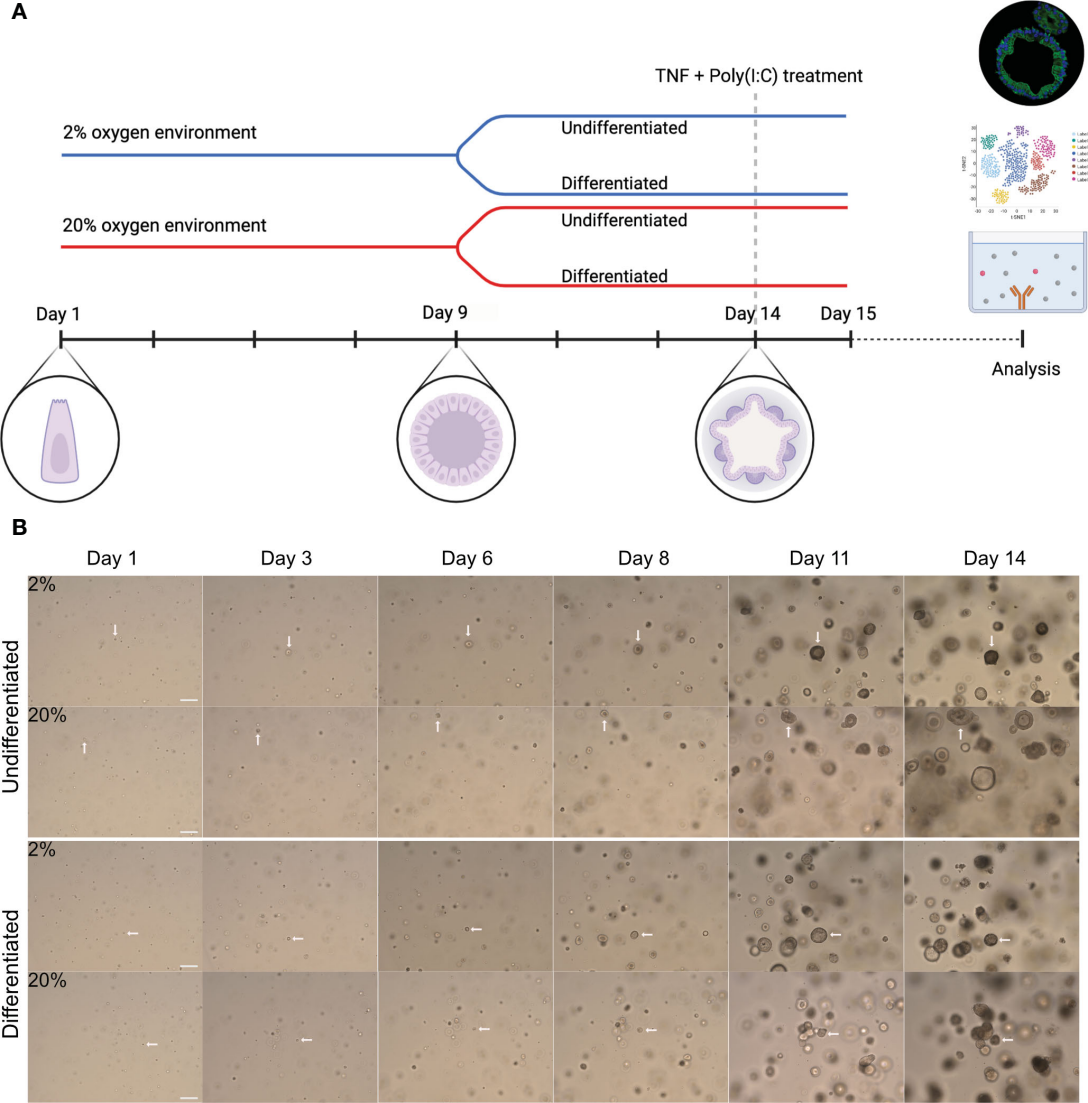
Experimental overview and growth of colonoids cultured in high and low oxygen. **(A)** General graphic representation of the experimental design. Colonoids were dissociated into single cells before plated at a density of 8000-10000 cells/50 ml matrigel and cultured in parallel in two incubators with 2% or 20% oxygen. Complete growth medium (CGM) was added every other day from establishment until 9 days, including ROCK inhibitor Y-27632 for the first two changes. On day nine (unless specified otherwise), cell differentiation was induced in half of the wells by replacing CGM with differentiation medium, while the other half continued with CGM. The colonoids were kept in culture until day 14 unless they were treated with pro-inflammatory TNF + poly(I:C). The blue line represents colonoids cultured in a 2% oxygen environment, while the red line represents cultivation in a 20% oxygen environment. As illustrated, stem cells began as single cells, proliferated into spheroids, and ultimately differentiated into 3D structured colonoids with crypts and a central lumen. For the stimulation assays, the colonoids were cultured as described until day 14. Subsequently, they were treated with TNF + poly(I:C) for 24 hours before the material (RNA and conditioned medium) was collected. **(B)** Representative brightfield images (10x magnification, EVOS microscope) of colonoid growth. The four image rows represent the four different conditions (undifferentiated or differentiated in 2% and 20%) cultured in parallel. The day the image was captured is indicated above the images. The white arrows follow a cell from a single cell to a 3D colonoid. Scale bar = 100 μm.

The general experimental design is illustrated in **Figure 1A**. For each independent experimental replicate, the colonoids were cultured in parallel in two separate incubators (New Brunswick Galaxy 170R CO2, Eppendorf, Hamburg, Germany) at 37°C with 5% CO_2_ and 2% or 20% oxygen. The oxygen concentration was lowered to 2% by calibrating the nitrogen input. The colonoids were cultured in CGM for the first 9 days (unless otherwise specified). Thereafter, half of the colonoids were differentiated, while the other half continued with growth medium and thus remained undifferentiated. Differentiation was induced by lowering Wnt-3A concentration to 5%, withdrawing Nicotinamide and SB202190 factor from the CGM, and adding the pan-Notch inhibitor DAPT (4.324 μg/mL, #2634, Bio-Techne). On day 14, A-83-01 was removed from the differentiation media before the TNF + Poly(I:C) stimulation assays (described below). Medium change was performed every two to three days until the end of the experiment. An overview of each donor and in which analyses they were included is found in **Table 1**.

**Table 1:** Colonoid donor characteristics and experimental analysis.

Donor (D)	D1 HC	D2 HC	D3 HC	D4 UC	D5 HC	D6 HC	D7 UC	D8 HC	D9 UC
Age	21	73	55	18	28	65	20	43	29
Sex	F	M	F	M	F	F	M	F	F
Growth analysis		x	x	x	x	x	x	x	
Cell markers	x	x	x	x	x	x	x		
Bulk RNA seq.^1^	x	x	x						
ScRNA-seq.	x								
NGAL ELISA			x	x	x	x	x		x
Multiplex			x	x	x	x	x		x

IBD, Inflammatory bowel disease; M, Male; F, Female; UC, Ulcerative colitis; HC, healthy control (non-IBD); RNA-seq, RNA sequencing; ScRNA-seq, Single-cell RNA-sequencing; NGAL, Neutrophile gelatinase-associated lipocalin.

The number of independent experimental replicates is listed in the figure legend.

1: A previously performed bulk RNA-sequencing (GSE172404) was also analyzed where a group of colonoids (n = 6) was cultured in 20% oxygen for 15 days, while another group (n = 6) was cultured in 20% oxygen except for the last 40 hours where they were cultured in 2% oxygen (38).

### Brightfield image analysis and linear mixed model of colonoid growth

During colonoid culture, brightfield images were acquired from each well with an EVOS microscope (Thermo Fischer Scientific) every two to three days, using 4X and 10X objectives to assess the total colonoid area. Images containing air bubbles were excluded (**SF1**, sheet 3). The images were analyzed *in silico* through Fiji using batch processing (39). The brightfield images (6-18 per condition) were converted into 8-bit images, and the auto local threshold function “Phansalkar” (radius = 10) was used to threshold the images. A region of interest was selected around the Matrigel-covered region. The “Particle analysis” function measured the area within the region of interest covered by colonoids as a proxy for cell mass. The settings for the particle analysis function included measuring particles from “10μm to infinity”, “excluding particles at the edges,” and “filling in holes.”

The experimental design had a hierarchical structure where every independent experimental replicate had several conditions with multiple technical replicates acquired at different time points. Thus, a linear mixed model (LMM) was utilized to evaluate colonoid growth statistically:

Formula = log(cell mass) ~ Day*factor(*Oxygen concentration*) + *Condition* + (1|*Donor*) + (1|*Day*)

The analysis was performed in R using the Lme4 (40) and LmerTest (41) packages. Day of image acquirement (1|*Day*) and independent experimental replicates (1|*Donor*) were introduced as random effects, (i.e., variability that might be present but not of interest). The fixed effects in the model were time (*Day*), the oxygen environment (*Oxygen concentration*), whether the colonoids were undifferentiated or differentiated (*Condition*), and the interaction between day and oxygen concentration (Day*Oxygen concentration), thus investigating if the *Day* variable changed the *Oxygen concentration* variable’s influence on colonoid growth. The model’s residuals followed a normal distribution.

### Immunofluorescence staining and confocal imaging of colonoids

Colonoids were collected for immunostaining at the end of the experiments (**Figure 1A**). In brief, colonoids from each experimental condition were pooled (n=3-6 wells) and resuspended in 50 μL Richard-Allan Scientific™ HistoGel™ Specimen Processing Gel (#HG-4000-012, Thermo Fisher Scientific, Waltham, MA), then fixed in 10% buffered formalin for 24-48 hours before they were embedded in paraffin as described elsewhere (42). Formalin-fixed paraffin-embedded sections were deparaffinized, and antigen retrieval was accomplished by boiling the sections in citrate buffer (pH 6.0) or Tris-EDTA buffer (pH 9.0) for 15 minutes in a commercial microwave oven. The sections were blocked with tris-buffered saline (TBS) + 5% bovine serum albumin (BSA) for five minutes. Primary antibodies against KI67 (Dako Agilent, Santa Clara, CA), Cytokeratin 20 (CK20) (Dako Agilent), Mucin-2 (MUC2) (Abcam, Cambridge, Great Britain), and Chromogranin A (CGA) (Abcam) were diluted in TBS + Tween^®^ 20 + 1% BSA and incubated overnight at 4°C (SF 1, sheet 2). The secondary staining was performed with the MaxFluor immunofluorescence detection system (MaxVision Bioscience, Ontario, Canada). Lastly, the sections were counterstained with DAPI (Thermo Scientific) and mounted with Glycergel (Dako Agilent).

### Confocal imaging of colonoids

Immunofluorescent images of the colonoids were captured with a confocal microscope (LSM 880 Airyscan, ZEISS, Oberkochen, Germany). Every image was captured at 20X magnification with standardized settings normalized to the section with the highest immunofluorescent expression (**SF1**, sheet 4). Five to ten images per section from eight independent experimental replicates were acquired.

### Immunofluorescent image analysis and quantification of cell markers in colonoid sections

Quantification of the cell markers was performed with three different methods depending on the distribution and expression of the proteins: 1) For MUC2 and CGA, the positive cells were manually counted; 2) KI67 was quantified by thresholding the images with “Otsu” in Fiji, and the number of KI67 positive cells was calculated with the “Particle analysis” function; 3) CK20 was processed through Fiji using the “HiLo” lookup table to reduce background noise while preserving the integrity of the signals from the colonoids. Then the “Measurement” function was used to measure each image’s signal intensity, resulting in an Integrated Density Score. The background of each image was measured independently three times, and the mean background was subtracted from the Integrated Density score. Thus, the corrected total cell fluorescence (CTCF) was calculated. The result from each quantification method was divided by the number of DAPI-positive cells present in each image to normalize the fluorescent expression to the number of cells. DAPI positive cells were quantified by thresholding the images with “Otsu” applying the “Watershed” and “Particle analysis” functions. The quantification scores are found in **SF1**, sheets 5-8.

### Bulk RNA-sequencing, enrichment analysis, and supervised datasets

RNA was extracted from the colonoids with the RNeasy mini kit (Qiagen, Hilden, Germany) per the manufacturer’s protocol, as previously described (15). Sequencing libraries were generated with SENSE total RNASeq library prep kit with RiboCop rRNA depletion (Lexogen GmbH, Vienna, Austria). The sequencing included 75 cycles of single-end reads conducted with the Illumina HiSeq4000 (Illumina, Inc., San Diego, CA, USA). FASTQ files were produced with bcl2fastq 2.18 (Illumina). LIMMA linear models identified differential gene expression between conditions with least square regression and empirical Bayes moderated *t* statistics. Correction-adjusted P-values ≤ 0.05 with Benjamin-Hochberg’s false discovery rate were statistically significant.

For the dataset GSE217663, RNA was extracted from untreated undifferentiated and differentiated colonoids cultivated at 20% oxygen (n= 3 donors) (**SF1** sheet 9). A supervised dataset of cell marker genes was created by searching the PangloaDB database (43) and used to identify differentially expressed cell markers in undifferentiated and differentiated colonoids. The cell marker gene list includes gen-sets for stem-, goblet-, absorptive-, enteroendocrine-, and tuft cells (**SF1**, sheet 10). It was used to identify cell clusters in the single-cell RNA-sequencing (scRNA-seq) dataset described below.

We also re-analyzed the in-house bulk RNA-seq dataset GSE172404, comparing fully differentiated colonoids cultivated continuously at 20% oxygen with colonoids cultivated at 2% oxygen for the last 40 hours, as previously described (38). Hypoxia-related genes were selected based on 1) Gene ontology (GO) term “cellular response to hypoxia” (GO:0071456, with applied filters: protein, homo sapiens. n=196 unique protein-coding genes) using the Amigo2 tool (version 2.5.12) gene ontology platform (44–46), downloaded 24.09.2019 and 27.03.2020 (DOI 10.5281/3727280); 2) HIF-1 downstream targets (n = 98 genes) as reviewed by Slemc and Kunej (47); 3) process network in MetaCore™ version 19.4 build 69900 “Transcription_HIF-1α targets” downloaded 01.04.2020 (n = 95 genes); and 4) individually investigated genes with experimental data indicating a connection to the cell’s response to hypoxia (n = 44 genes), resulting in a list of 369 unique genes (**SF1**, sheet 11).

### Single-cell RNA-sequencing, bioinformatics, and enrichment analysis

Single cells were processed through the 10x Genomics Chromium Single Cell Platform (Single Cell 3’ v3) (pipeline version 3.1.0) (10x Genomics, Pleasanton, CA). In brief, differentiated and undifferentiated colonoids grown in 2% and 20% oxygen were enzymatically (Trypsin + Y-27632 for 10 minutes at 37°C) and mechanically (18G needle) dissociated into a single cell suspension consisting of Phosphate-Buffered Saline (PBS) + 0.05% BSA. Ten thousand cells were loaded onto the platform for each sample. Single-cell sequencing data was analyzed using the 10x Genomics Cellranger software (version 3.1.0). In total, 13904 cells were successfully sequenced (see **Table 2**). The BCL files were converted to FASTQ format and mapped to the GRCh38 reference genome. Cellranger was further used to generate UMI counts from those droplets likely to contain at least one cell and aggregated into a read depth normalized feature count matrix. The 10x Genomics cellbrowser (Cloupe version 5) was used for visualization purposes and cell type assignment of the aggregated data. Downstream analysis was conducted primarily using the Seurat R package (48). Cells with low counts (<200) or classified as low quality by the miQC R package were excluded (49). Features with counts lower than 500 or higher than 40000 were also excluded. The count data was further normalized with the transform varians accounting for the mitochondrial fraction, and the top 3000 highly variable genes were used for principal component analysis (PCA) dimension reduction. The top 40 principal components were used as input to the uniform manifold approximation (UMAP) method and clustered using the “FindClusters” function. The cell-type classifications were refined to match the clustering output, and differential expression between groups was identified with the non-parametric Wilcoxon rank-sum test using the Seurat function “FindMarkers.”

**Table 2:** Single-Cell RNA-sequencing.

Sample	indentsecretEstimated number of cells	Mean reads per cell	Median genes per cell
2% undifferentiated	5219	21539	2356
20% undifferentiated	4476	22946	2648
2% differentiated	2111	50602	3092
20% differentiated	2098	63352	3560

Enrichment analyses were performed with Metacore (Clerviate, London, Great Britain) to compare cell clusters cultured in 2% *vs*. 20% oxygen. Gene lists were created in R with the Seurat package for the stem-like, progenitor, and differentiated cell clusters, including a prefiltering process (logfc.threshold = log (2), min.pct = 0.25 and min.diff.pct = 0.1).

### Multiplex chemokine profiling and ELISA

Colonoids were treated with TNF (100 ng/mL, PeproTech) + Poly(I:C) (20 μg/mL, *In vivo*Gen, San Diego, CA) on day 14 for 24 hours to investigate chemokine and Neutrophil Gelatinase-Associated Lipocalin (NGAL) secretion (**Figure 1A**). The analyzed samples included four conditions (2% oxygen untreated, 2% oxygen treated, 20% oxygen untreated, and 20% oxygen treated) from six independent experimental replicates (**Table 1**). The conditioned medium was collected, frozen at -80°C, and later thawed for analysis. The Bio-Plex Pro Human Chemokine Panel, 40-plex (Bio-Rad Laboratories, Hercules, CA, United States), was used to analyze undiluted samples according to the manufacturer’s instructions using the Bio-Plex 200 Systems.

Per the manufacturer’s protocol, a sandwich ELISA kit (R&D Systems, Minneapolis, MN) was used to measure the NGAL concentration within the conditioned media. The samples were analyzed in duplicates with a working dilution of 1:50 and 1:100. Shortly, the capture antibody was diluted in PBS and incubated overnight at room temperature (RT) in 96 well plates. The plates were washed three times with PBS + 0.05% Tween^®^ 20 before being blocked with Reagent diluent (1% BSA in PBS) for one hour at RT. The samples were distributed and incubated for two hours at RT before detection antibody was added and incubated for two hours in RT. The plates were removed from light sources, incubated with Streptavidin-HRP (1:40), and then substrate solutions for 20 minutes. Lastly, a stop solution (2N H_2_SO_4_) was added, and the plates were immediately analyzed with a microplate reader (iMark, Bio-Rad, Hercules, CA).

### Figures

Graphs were created using the R packages ggplot2 (50) and Seurat (48), and GraphPad Prism 9.0 (GraphPad Softwear Inc., San Diego, CA).

### Statistical analysis

Statistical analyses, excluding bioinformatical analysis of sequencing data and the linear mixed model (LMM) of colonoid growth described above, were conducted in GraphPad Prism 9.0. The Shapiro-Wilk test was used to determine normal distribution. Cell marker data were analyzed with Wilcoxon’s test and one-way ANOVA followed by the Šídák test for multiple comparisons. The chemokine and ELISA data were analyzed with repeated measures (RM) one-way ANOVA followed by Šídák’s multiple comparisons test. Normally distributed paired groups were analyzed with Paired t-test. Non-parametric analyses with two paired groups were performed with the Wilcoxon test. P-values < 0.05 were considered statistically significant.

### Ethical considerations

The current study was carried out under relevant approvals by the Central Norway Regional Committee for Medical and Health Research Ethics (reference numbers 5.2007.910, 134436 and 2013/212/REKmidt). All patients included in the study provided informed written consent.

## Results

### A low oxygen environment is beneficial for colonoid growth

**Figure 2:**
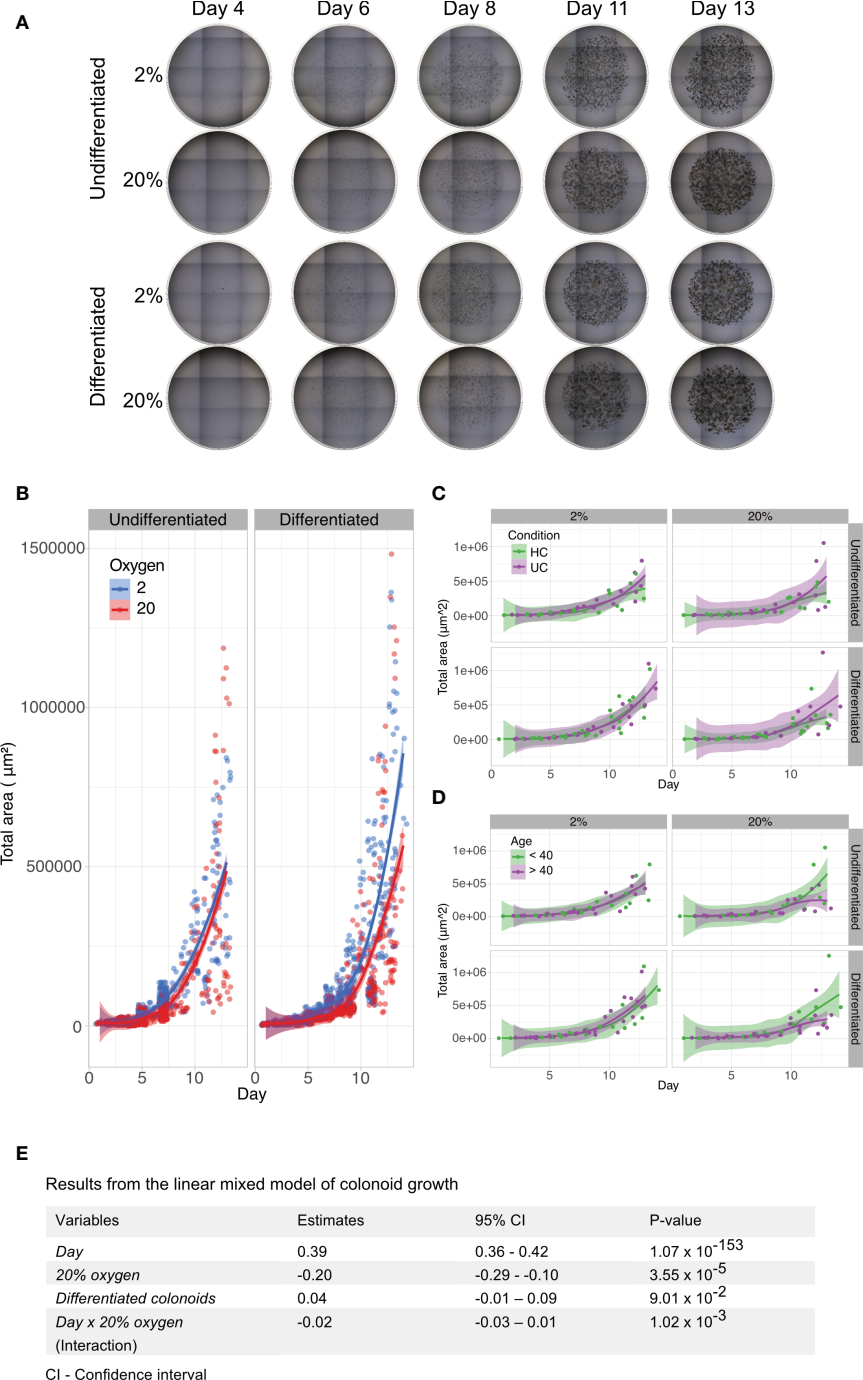
Brightfield image analyses and linear mixed model of colonoid growth. **(A)** Two dimensional (2D) brightfield images (4x magnification, EVOS microscope) of Matrigel domes containing undifferentiated or differentiated colonoids cultured in parallel at 2% and 20% oxygen. Nine tiled images captured every dome within one technical replicate (i.e., plate well) throughout the experiment. The day of image acquisition is indicated above the images; the oxygen environment and the differentiation status are shown on the left. **(B)** Computational growth analysis of colonoid cultures (n=11 independent experiments, with 6-12 technical replicates (i.e., plate wells) per condition. **Table 1** and **Supplementary Figure 2B**). Days are marked on the x-axis, while the y-axis shows the total colonoid mass in 2D. Timepoints with blue circles represent 2% oxygen cultivation, and red circles represent 20% oxygen cultivation in undifferentiated (left panel) and differentiated (right panel) colonoids. The blue (2% oxygen) and red (20% oxygen) lines were fitted with the Loess curve fitting model. **(C)** Colonoid growth curve of ulcerative colitis (n=2) and healthy control donors (n=5), with data from independent experiments with the same donor merged). The x-axis represents time, while the y-axis shows the total colonoid mass in 2D. Timepoints with purple circles represent ulcerative colitis donors, and green circles represent healthy control donors. Undifferentiated colonoids (left panel) and differentiated (right panel). The purple (ulcerative colitis) and green (healthy controls) lines were fitted with the Loess curve fitting model. **(D)** Colonoid growth patterns of donors < 40 years (n=3) and > 40 years (n=4)). The x-axis represents time, while the y-axis shows the total colonoid mass in 2D. Timepoints with purple circles represent old donors, and green circles represent young donors. Undifferentiated colonoids (left panel) and differentiated (right panel). The purple (> 40) and green (< 40) lines were fitted with the Loess curve fitting model. **(E)** A linear mixed model based on data from 1875 images was used to quantify which variables impacted colonoid growth. In the table, fixed variables are listed in the first column. The middle columns are absolute values of how much the variables affected growth, with an estimate of the impact in the left-middle column and 95% confidence intervals in the column to the right. P-values are listed in the last column.

To investigate whether colon-derived stem cells could establish and grow in a low physiologic oxygen environment *ex vivo*, we cultured colonoids at 2% and 20% oxygen in parallel (**Figure 1A**). Brightfield images were captured every two to three days following colonoid development (**Figure 1B**). Single stem cells established into colonoids in both 2% and 20% oxygen environments. As outlined by the white arrows in **Figure 1B**, colonoid growth was initially slow, followed by a rapid expansion around day six at both oxygen conditions. We then assessed the growth process *in silico*. The computational analysis included converting brightfield images to binary images and measuring the total cell mass present in the picture as a proxy for cell quantity and size. In total, 1875 images were acquired from 11 independent experiments (**Table 1** and **Supplementary Figure 2B**) with six to twelve technical replicates (i.e., plate wells) per condition (i.e., 2% undifferentiated, 2% differentiated, 20% undifferentiated, and 20% differentiated) (**Figure 2A**). The image analysis showed that the growth curve of the colonoids was equal to classical cell growth curves with an initial lag phase followed by an exponential phase where the cell mass increased rapidly (**Figure 2B** and **Supplementary Figure 2**). Since the assays ended on day 14, the death phase did not become apparent. Cultivation at 2% oxygen did not impede colonoid growth (**Figure 2B**). For differentiated colonoids, the growth appeared enhanced at 2% oxygen. Since we observed experiment-to-experiment variations, we investigated if the donor’s IBD status or age (**Table 1**) affected the colonoids growth patterns. Interindividual differences were more prominent than disease status (**Figure 2C**) and age (**Figure 2D**), as shown by overlapping CI between the groups. However, it may be of interest for further studies that colonoids from younger donors (<40 years) appeared to be larger in 20% oxygen than colonoids from older donors(<40 years).

An LMM was performed for statistical information about variables that affected cell growth (**Figure 2E**). The variables included time (*Day*), the oxygen environment with 20% as a baseline (*20% oxygen*), whether the colonoids were undifferentiated or differentiated (*Differentiated colonoids*), and the interaction between time and oxygen concentration (*Day* x *20% oxygen*). The most important variable for colonoid mass was the time (P-value < 0.0001, confidence interval (CI) 0.36 to 0.42). The total cell mass increased every day. Culturing colonoids in 20% oxygen had a statistically significant negative impact on colonoid growth (P-value < 0.0001, (CI) -0.1 to -0.29). The result applied to the group of independent experimental replicates together, although there were interindividual variations (**Supplementary Figure 2B**). As previously noted, the effect of the oxygen environment was more pronounced from day six. The LMM supports this because the interaction variable between time and oxygen concentration had less impact on growth than oxygen alone. How much the oxygen environment affected total cell mass depended on when the comparison was performed. Between oxygen concentrations, the disparity in cell mass was less pronounced on day two than on day twelve (e.g., Donor 8_A, **Supplementary figure 2B**). The interaction effect had a significant P-value, although with a CI overlapping zero (P-value 0.001, CI -0.03 to 0.01). Whether the colonoids remained undifferentiated or differentiated did not impact total cell mass. Overall, we found that human colonoids grew similarly or even better in a 2% oxygen environment as in a 20% oxygen environment (**Figure 2E**).

### Colonoids proliferate and differentiate into specialized cell types in both 2% and 20% oxygen

**Figure 3:**
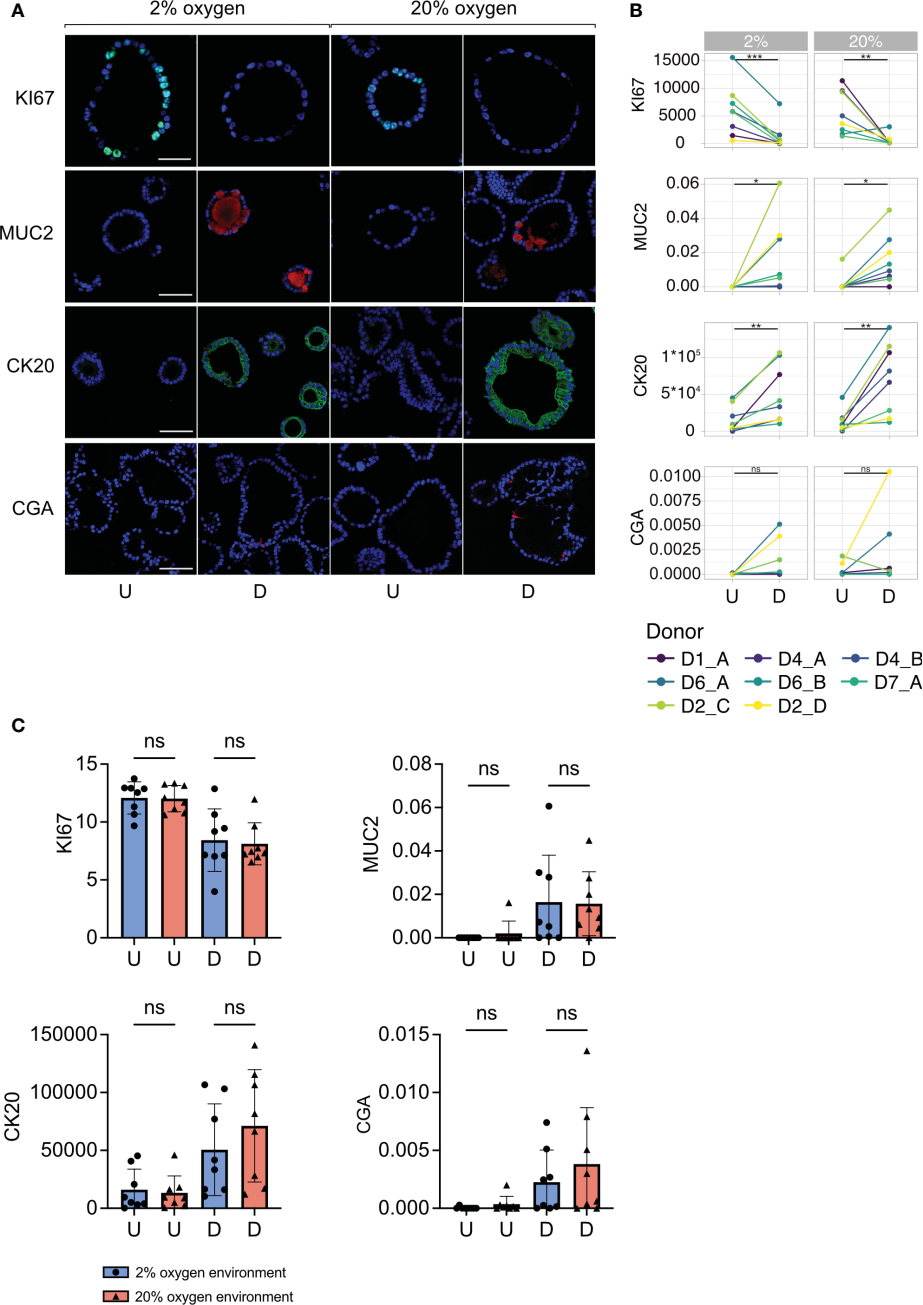
Cell marker protein expression in undifferentiated and differentiated colonoids at low and high oxygen. **(A)** Immunofluorescent images of cell marker expression. Images in the two first columns show colonoids cultured in 2% oxygen, while the images in the last two columns show colonoids cultured in 20% oxygen. Within each oxygen concentration, U indicates the undifferentiated condition, while D indicates the differentiated condition. Each row represents a cell marker protein. DAPI was used as a counterstaining in all images. Scalebar = 50 μm. **(B)** Paired quantification of cell marker expression in undifferentiated (U) and differentiated (D) colonoids (n=8 independent experiments, **Table 1**). Statistical testing was carried out with the Wilcoxon test. The x-axis shows the differentiation condition of the colonoids while the y-axis shows calculated expression: CK20 and KI67 expressions were quantified with corrected total cell fluorescence (CTCF) adjusted for the number of cells present. The y-axis for MUC2 and CGA indicates the fraction of positive cells, manually counted, among all present cells. **(C)** Cell marker protein expression in colonoids cultured at 2% *vs*. 20% oxygen. The bars show the mean and standard deviation with each independent experiment plotted as individual values. Blue bars and dots illustrate colonoids cultured in 2% oxygen and red bars and triangles cultured in 20% oxygen. The x-axis shows the conditions undifferentiated (U) or differentiated (D), while the y-axis shows cell marker expression. P-values are determined by one-way ANOVA followed by Šidák’s multiple comparisons test. ns = non-significant.

A hallmark of human-derived colonoids is their ability to mimic the cellular composition of the human colon epithelium (42). We investigated whether the same cell types were present in colonoids cultured in 2% and 20% oxygen. In eight independent experiments (**Table 1**), colonoids were cultured at 2% and 20% oxygen in parallel and divided into undifferentiated and differentiated colonoids (**Figure 1A**). Sections of paraffin-embedded colonoids were stained for KI67, MUC2, CK20, and CGA, to detect cells with proliferation potential, goblet cells, differentiated epithelial surface cells, and enteroendocrine cells, respectively. Images of the sections were captured with a confocal microscope (**Figure 3**), as described in the method section.

First, we investigated differences in cell marker expression between undifferentiated and differentiated colonoids. The expression of KI67 was significantly higher in undifferentiated compared to differentiated colonoids (**Figures 3A, B**, row 1) (P-value = 0.0008 for 2% and 0.003 for 20%). The undifferentiated colonoids expressed almost no MUC2 (**Figures 3A, B**, row 2). Differentiated colonoids expressed MUC2 in addition to the semilunar cell nuclei characteristic for goblet cells. The expression of MUC2 was significantly higher in differentiated *vs*. undifferentiated colonoids (**Figure 3B**, row 2) (P-value = 0.03 for 2% and 0.02 for 20%). CK20 expression was significantly increased in differentiated compared to undifferentiated colonoids (**Figures 3A, B**, row 3), (P-value = 0.009 for 2% and 0.004 for 20%). Co-staining of CK20 and MUC2 showed numerous cells positive for CK20 but negative for MUC2 (**Supplementary Figure 1**). Since absorptive cells are the most numerous in the colonic epithelium and simultaneously do not express MUC2, these CK20 positive, MUC2 negative cells probably represent absorptive cells. CGA was present in some cells within the differentiated colonoids but not in undifferentiated colonoids (**Figure 3A**, row 4), except for some expression in two independent replicates from D2 cultured in 20% oxygen (**Figure 3B**, row 4). A unique feature of colonic enteroendocrine cells is their cellular processes extending into the lumen (51). This quality is also present in colonoid enteroendocrine cells, as depicted in **Figure 3A**, row 4. Due to the low number of cells expressing CgA, the increase in differentiated compared to undifferentiated colonoids was marginally non-significant (P-values for 2% and 20% = 0.06). In brief, cell markers for post-mitotic intestinal epithelial cells (IECs) had an increased expression in differentiated colonoids. IECs with a potential for proliferation were more abundant in undifferentiated colonoids. Whether the oxygen environment affects stem cell differentiation in colonoids is not previously described. Therefore, we compared the expression of cell markers across oxygen conditions. There were no significant differences in the expression of KI67, MUC2, CK20, or CGA between colonoids cultured in 2% and 20% oxygen environments (**Figure 3C**). Overall, this suggests that the oxygen environment does not influence the colonoid’s IECs composition.

### Single-cell RNA-sequencing shows similar cell clusters in 2% and 20% oxygen

**Figure 4:**
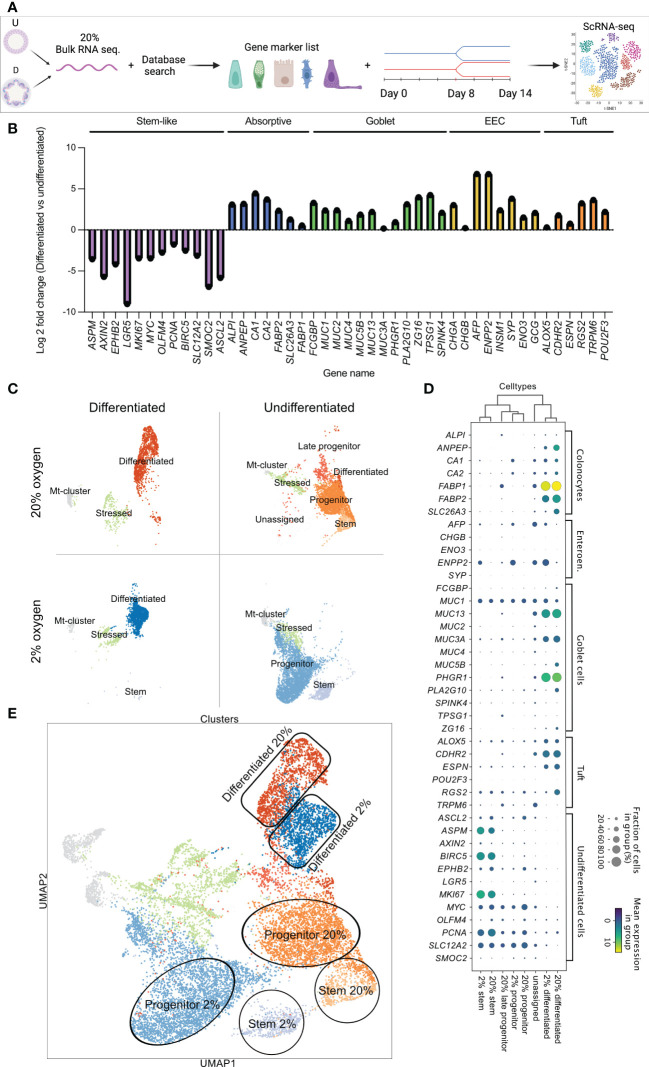
Identification of cell marker genes in undifferentiated and differentiated colonoids by bulk RNAseq **(B)** and single-cell RNAseq. **(C-E)**. **(A)** Schematic diagram of the experimental workflow. **(B)** Cell marker gene expression in differentiated *vs*. undifferentiated colonoids cultured in 20% oxygen. PangloaDB database (43) was used to identify cell marker genes, as described in the Method section. The x-axis shows gene names, and the y-axis shows the log2 foldchange for indicated genes in differentiated compared to undifferentiated colonoids (average of n=3). Cell marker genes are grouped and colored by cell type; purple for stem cells, blue for absorptive cells, green for goblet cells, yellow for enteroendocrine, and orange for tuft cells. **(C)** Visualization plot of scRNA-seq data from 13904 single cells. Four culture conditions (differentiated and undifferentiated in 2% and 20% oxygen) were analyzed in separate batches represented by their own UMAP-plot. The gene list confirmed in Bulk RNAseq **(B)** and **SF1**, sheet 10 was used to identify and annotate each cluster. **(D)** Dot plot showing expression of cell marker genes in the different cell clusters. Cell clusters are listed along the x-axis, while cell marker genes are listed along the y-axis. The color intensity represents mean expression within a cluster, and the radius of the dot represents the fraction of cells in the cluster expressing a gene. **(E)** Combined UMAP-plot for all four conditions. Clusters with equal annotations are illustrated by the circles (Stem cells), rectangles (Differentiated cells), and oval shapes (Progenitor cells) and color-coded as in 4C.

Our data showed that culturing colonoids in a low oxygen environment is possible, without differences in KI67, MUC2, CK20, or CGA protein expression between physiological hypoxia (2% oxygen) and supraphysiological 20% oxygen level. However, whether there are any alterations in the transcriptome of the different cell types when culturing colonoids in a low oxygen environment remains unknown. Thus, we performed scRNA-seq to investigate cell-dependent transcriptomic alterations between colonoids cultured in 2% *vs*. 20% oxygen (**Figure 4**). To annotate the cell clusters present within the scRNA-seq, we first created a supervised gene list by searching for gene markers in the PangloaDB (43). The regulation of the cell marker genes was confirmed in an in-house bulk RNA-seq dataset of undifferentiated *vs*. differentiated colonoids cultured in 20% oxygen (**SF1**, sheet 9). Lastly, the gene list was utilized to annotate the cell clusters of the scRNA-seq (see the experimental outline in **Figure 4A**). Bulk RNA-seq of differentiated and undifferentiated colonoids (n=3) cultured at 20% showed that genes characteristic for stem and progenitor cells (*ASPM, AXIN2, WPHB2, LGR5, MKI67, MYC, OLFM4, PCNA, BIRC5, SLC12A2, SMOC, ASCL2*) were upregulated in the undifferentiated colonoids. Genes characteristic for absorptive colonocytes (*ALP1, ANEP, CA1, CA2, FABP2, SLC26A3, FABP1*), goblet- (*FCGBP, MUCs 1, 2, 5, 3B, 13, 3A, PHGR1, PLA2G10, ZG16, TPSG1, SPINK*), enteroendocrine- (*CHGA, CHGAB, AFP, ENPP2, INSM1, SYP, ENO3, GCG*),- and tuft cells (*ALOX5, CDHR2, ESPN, RGS2, TRPM6, POU2F3*) were upregulated in the differentiated colonoids (**Figure 4B**).

**Figure 5:**
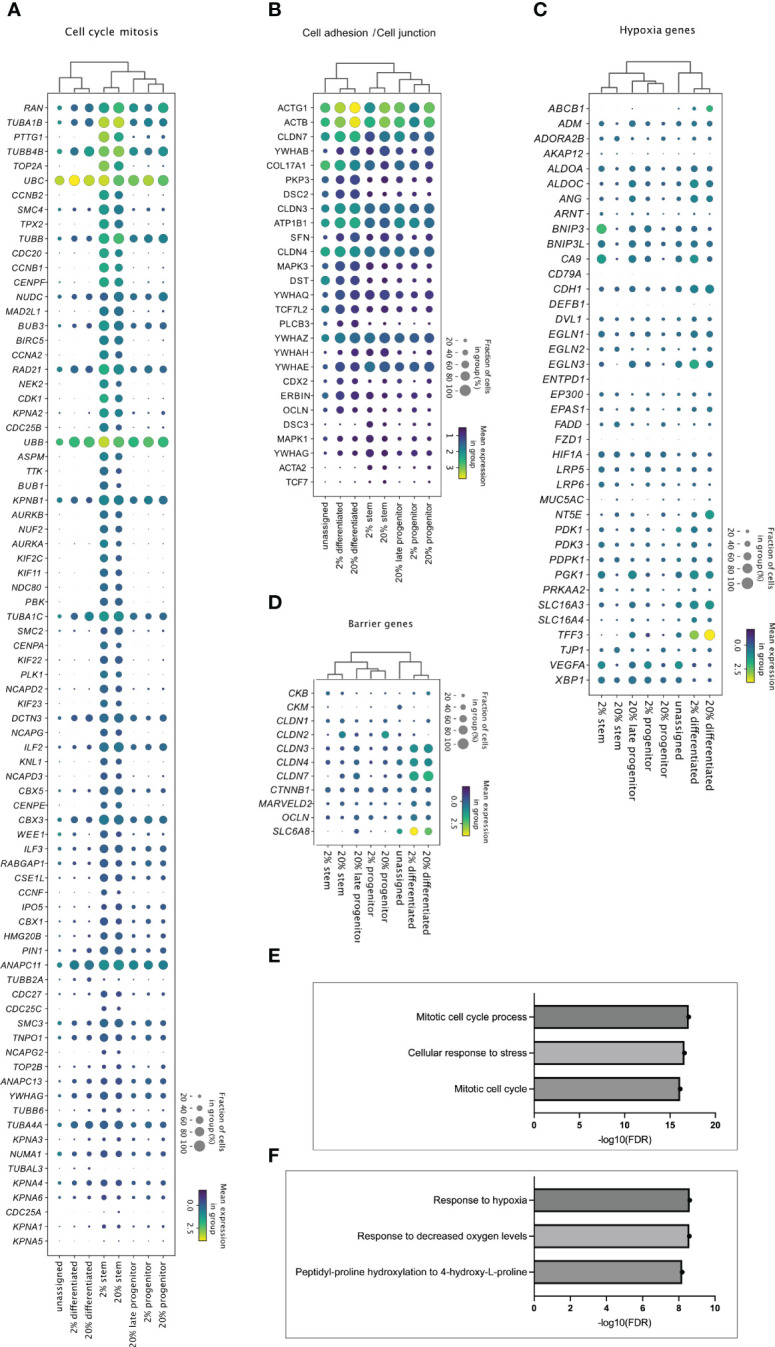
Cell cluster specific transcriptome in 2% and 20% oxygen. Enrichment analysis of upregulated genes in the stem, progenitor, and differentiated cell clusters from 2% *vs*. 20% oxygen culture (adjusted P <0.05). were generated with MetaCore+MetaDrug™ version 21.3 build 70600 (**SF1**, sheets 12-15). The dot plots display how **(A)** genes related to cell cycle and mitosis **(A)**, genes associated with cell adhesions and cell junctions **(B)**, hypoxia-related genes **(C)** and barrier genes **(D)** were expressed in the different cell type clusters. Cell clusters are listed along the x-axis, while genes are listed along the y-axis. The color intensity represents mean expression within a cluster, and the radius of the dot represents the fraction of cells in the cluster expressing a gene. **(E, F)** Bars indicate –Log10 false discovery rate (FDR), while the y-axis lists top three GO processes upregulated in stem-like cell clusters **(E)** and differentiated cell cluster **(F)** in 2% compared to 20% oxygen culture (**Supplementary file SF1**, sheets 14 and 15, respectively).

For the scRNA-seq, four samples of colonoids (2% undifferentiated, 2% differentiated, 20% differentiated, and 20% differentiated) were analyzed in separate batches (**Figure 4A**). The cell marker gene list confirmed by the bulk RNA-seq (**Figure 4B**) was utilized to separately characterize the cell clusters present in each UMAP-plot (**Figure 4C**). Furthermore, a supervised comparison of the genes regulated within each cluster was performed. In both 2% and 20% oxygen, differentiated cell clusters were present in the differentiated colonoids (first column of **Figure 4C**) and stem cell clusters and progenitor cell clusters were present in the undifferentiated colonoids (second column of **Figure 4C**). Within the differentiated clusters, the cells overlapped in their expression of cell type genes for absorptive-, goblet-, enteroendocrine- and tuft cells. Thus, making it difficult to further divide the cluster into, e.g., absorptive- and goblet cells. Consequently, we annotated the cluster as differentiated cells. Undifferentiated colonoids cultivated at 20% oxygen had more cells sharing gene expression with differentiated cells (i.e., late progenitor and differentiated clusters in the second column of **Figure 4C**) than colonoids cultivated in 2% oxygen, indicating that auto-differentiation was more prominent at high than low oxygen level. In undifferentiated colonoids cultured at 20% oxygen we also observed some cells (“unassigned cluster”) that appear to be slightly more differentiated than late progenitor cells within the same culture (**Figure 4D** and **Figures 5A-D**). Every condition had an mt-cluster representing dead or severely stressed cells. The mt-clusters and stressed clusters were more numerous in the differentiated conditions compared to the undifferentiated conditions in both 2% and 20% oxygen.

Each cell type specific cluster shared the same signature genes when looking at the fraction of cells that expressed the gene and the mean expression in the group (visualized by dot-blot in **Figure 4D**). For instance, the differentiated cell clusters had similar expressions of *FABP1*, *MUC13*, or *PHGR1*. The stem cell clusters had similar expressions of *BIRC5, ASPM*, and *MKI67*. Mean expression and the fraction of cells expressing specific genes (e.g., *FABP1*, *BIRC5*, and *MUC13*) were equal in cell clusters originating from 2% and 20% oxygen environments. The main difference between stem cell clusters and progenitor cell clusters was that progenitor cell clusters had a lower expression of classical stem cell genes (e.g., *PCNA, SLC12A2*, and *MYC*) while also sharing gene expression characteristics for differentiated cells (e.g., *FABP1*, *CA1* or *ENPP2*). After each cluster was annotated, the cells were combined in a joint UMAP plot, where clusters with equal annotations in 2% and 20% oxygen merged as illustrated by the circles, rectangles, and oval shapes in **Figure 4E**. This observation suggests that the equal cell type clusters across oxygen environments share common characteristics. Overall, and corresponding to immunofluorescence data, the results from the scRNA-seq indicate that the oxygen environment does not alter the cell type composition of the colonoids.

### Transcriptomic differences among cell clusters in 2% and 20% oxygen

Distinct cell types are exposed to different oxygen concentrations within the colon, with stem cells at the bottom of the crypts being exposed to a higher concentration than apical differentiated IECs adapted to thrive in an oxygen environment close to 0% (23). Hence, of natural interest is how the transcriptome is regulated in undifferentiated and differentiated cells cultured in physiological hypoxia (2% oxygen) compared to supraphysiological 20% oxygen level.

Enrichment analysis showed that the stem-like cell clusters in both 2% oxygen (**SF1**, sheet 12) and 20% oxygen (**SF1**, sheet 13) had significant upregulation of gene network related to cell cycle and mitosis compared to progenitor and differentiated cells **Figure 5A**). Differentiated cell-clusters had upregulated networks related to e.g., cytoskeleton and cell junctions compared to stem and progenitor cells (**Figure 5B** and **SF1**, sheets 12-13). Thus, the cell clusters identified based on cell marker genes (**Figure 4**) showed molecular signatures (**Figure 5**) that characterize stem cell function and features in differentiated cells of the human colon epithelium (7, 52) Enrichment analysis was then performed on the genes regulated within the stem-, and differentiated cell clusters from 2% oxygen environment compared to 20% oxygen environments (**SF1**, sheets 14-15). In the stem cell clusters from colonoids cultivated at 2% oxygen, many GO processes related to the cell cycle were upregulated compared to the stem cell cluster from colonoids cultivated at 20% oxygen (**Figure 5E**, **SF1**, sheet 14). At 2% oxygen, the differentiated cell cluster had a significant HIF-1 response (**Figure 5F**) where canonical HIF-1 targets were upregulated, including *Carbonic anhydrase IX, Adiphophilin, GLUT1, Galectin-1 (Gal-1), P4HA1, VEGF-A, IBP3, Nip3, Nucleuphosmin* (as listed in SF1, sheet 15). Many of the hypoxia-related genes (**Figure 5C**) were upregulated in the differentiated clusters at both physiological hypoxia and supraphysiological oxygen, while for the stem- and progenitor clusters, the genes were mainly regulated in cells from colonoids cultivated at 2% oxygen. Since physical barrier protein expression is regulated by HIF-1α (25), and HIF-1α responses are altered in 2% oxygen, we next looked at how barrier function-related genes were regulated in the cell clusters (**Figure 5D**). We found that barrier-related genes were most prominently expressed in the differentiated cell clusters. The mean expression was similar in both oxygen environments, with the exemption of *SLC6A8*, which had a higher mean expression in the differentiated cluster from colonoids cultivated at 2% oxygen. In short, we have shown that cultivating colonoids in physioxia alters gene regulation with increased cell-cycle-related activity and HIF-1 target response.

### Differentiated colonoids respond to a pro-inflammatory signal in both 2% and 20% oxygen but are hyperresponsive in supraphysiological oxygen

**Figure 6:**
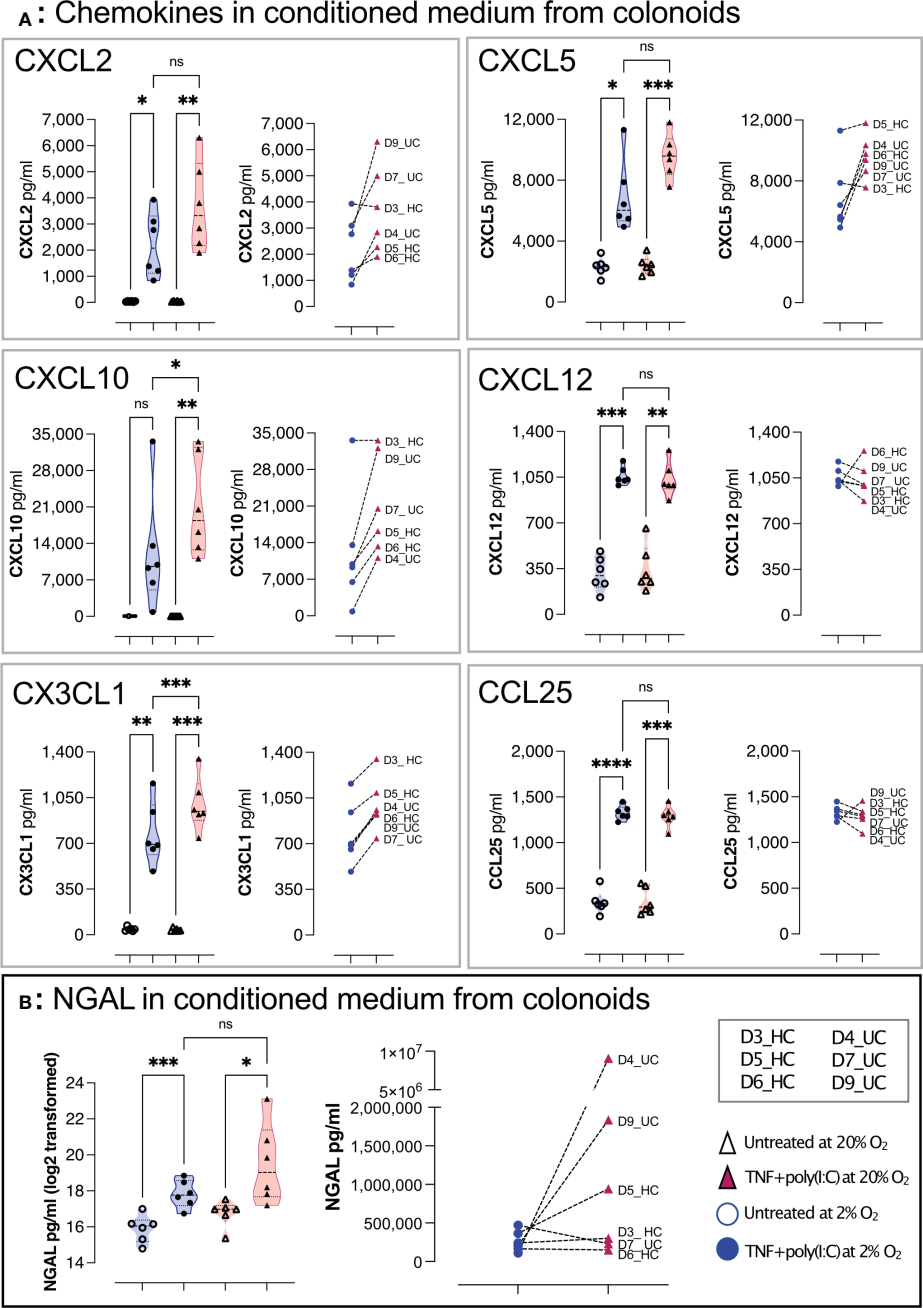
TNF+ Poly(I:C) induced chemokines and NGAL release from colonoids grown at high and low oxygen concentrations. Chemokines **(A)** and NGAL **(B)** in conditioned medium from differentiated untreated colonoids and after 24 hours of treatment with TNF + Poly(I:C). The treatment groups are represented on the x-axis, while the concentration of the target molecule is represented on the y-axis. In **(A)**, CXCL2, CXCL5, CXCL10, CXCL12, CX3CL1, and CCL25 concentrations (pg/mL) in conditioned medium were detected by Bio-plex Pro-Human Chemokine Panel analysis. In **(B)** NGAL was detected by ELISA. In each panel, the violin plots show the differences between untreated and TNF+Poly(I:C) treated colonoids cultivated at 2% oxygen (blue) and 20% oxygen (red). Each independent experimental replicate is plotted as individual values, and the graph to the right in each panel shows paired data for each donor treated with TNF+Poly(I:C) at 2% (blue circles) and 20% (red triangles). Statistical analyses were performed using RM one-way ANOVA followed by Šídák’s multiple comparisons test. In left panel **(B)**, NGAL was plotted and analyzed on log2 transformed data. Right panel **(B)** shows paired NGAL concentrations as pg/ml for each donor. See **Table 1** for colonoid donor characteristics * < 0.05, ** < 0.005, *** < 0.001, and **** < 0.0001. ns = non-significant.

Upon exposure to signals from microbiota and immune cells, or DAMPs from, e.g., dying and infected cells, the colonic epithelium secrete immunomodulators such as chemokines and NGAL that regulate homeostasis and inflammation in the gut (10, 42). NGAL is an acute-phase protein secreted from colonic epithelial cells during inflammatory colonic diseases (8, 10). Therefore, we next addressed whether chemokine and NGAL release were altered in colonoids cultured at 2% oxygen compared to colonoid cultured at 20% oxygen. We performed an assay treating differentiated colonoids grown at 2% or 20% oxygen with a synthetic analog of dsRNA associated with viral infections and DAMP (Poly(I:C)) together with pro-inflammatory Tumor necrosis factor (TNF) and analyzed the conditioned medium for chemokines and NGAL by multiplex chemokine assay and ELISA, respectively. TNF + Poly(I:C) induced significantly increased release of CXCL2, CXCL5, CXCL10, CXCL12, CX3CL1 and CCL25 from colonoids grown in low as well as high oxygen concentration (**Figure 6A**). We found significantly lower CXCL10 and CX3CL1 concentration in 2% *vs*. 20% oxygen, and there appeared to be a tendency for decreased pro-inflammatory response to TNF + Poly(I:C) in 2% oxygen for CXCL2 and CXCL5 for most donors. We found a statistically significant increase in NGAL secretion from TNF + Poly(I:C) treated colonoids in both 2% and 20% oxygen, but the experiment-to-experiment variation was larger in 20% than in 2% oxygen. The pro-inflammatory NGAL response was lower in 5 of 6 colonoid cultures at 2% oxygen compared to colonoids cultivated at 20% oxygen (**Figure 6B**, right panel). Thus, colonoids established and cultivated at 20% oxygen (i.e., hyperoxia) appeared hyperresponsive compared to colonoids cultivated at 2% oxygen (i.e., physiological hypoxia). This is in line with our previous findings that TNF/IL17 induced inflammatory-associated genes and release of chemokines were attenuated in differentiated colonoids cultivated in 2% oxygen for the last 40 hours [(38) and GSE172404].

### Short-time reduced oxygen levels alter the expression of genes related to differentiation, metabolism, mucus lining, and immune networks in differentiated colonoids

**Figure 7:**
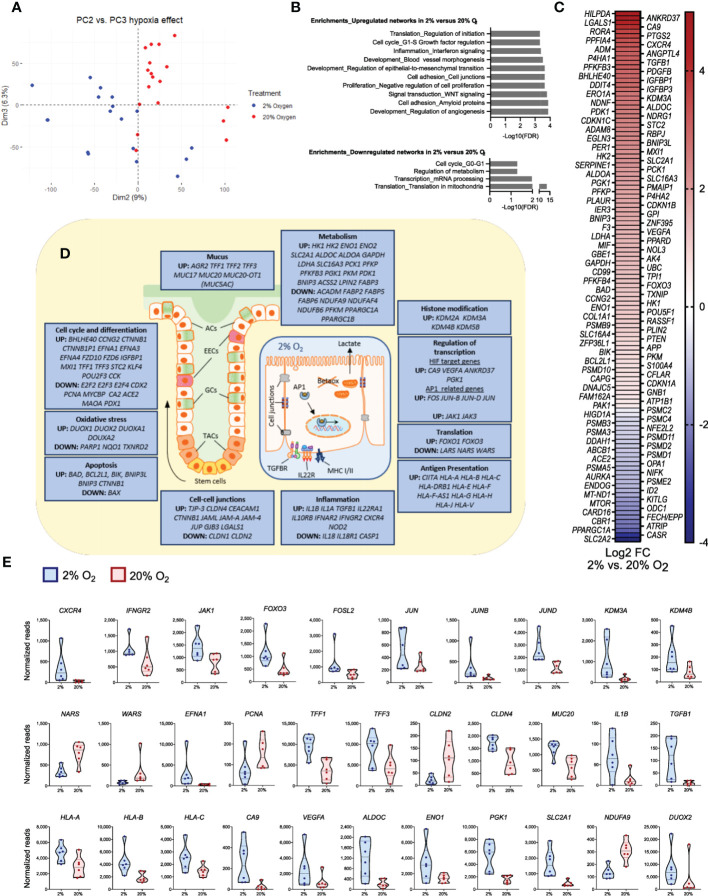
Significant changes in epithelial gene expression after a short time reduction of oxygen. The panels show data from bulk RNA-seq analysis of differentiated colonoids grown continuously at 20% or at 2% for the last 40 hours. **(A)** PCA plot of PC2 *vs*. PC3 for the complete dataset from GSE172404 (38) captures the influence of oxygen levels. Dots represent individual samples. **(B)** Enrichment analysis of basal gene expression in colonoids grown at 2% compared to 20% oxygen. Networks (top ten) best associated with upregulated genes (n=1192) and networks (top four) best associated with downregulated genes (n=1192), analyzed using MetaCore™ version 6.34 build 69200. The strength of association is given as -Log10 false discovery rate (FDR). **(C)** Differential expression of curated hypoxia-related genes (n=119) in 2% oxygen compared to 20% oxygen (adjusted P-values < 0.05). The heatmaps are sorted from highest (red) to lowest (blue) log2 fold change (FC) values, with 89 upregulated and 30 downregulated genes in 2% compared to 20% O_2_. **(D)** Illustrations of a colonic crypt with mucus lining and a colonic epithelial cell. The blue boxes contain a selection of differentially regulated genes between colonoids at 2% or 20% oxygen (adjusted P-values < 0.05), grouped by intestinal epithelial cell functions. Upregulated or downregulated genes at 2% compared to 20% oxygen are mentioned in boxes according to the topic. ACs, absorptive cells; EECs, enteroendocrine cells; GCs, goblet cells; TACs, Transit-amplifying cells; TGFBR, transforming growth factor beta receptor; IL22R, Interleukin 22 receptor complex; Betaox, beta-oxidation of fatty acids; MHC, major histocompatibility complex; AP1, network of transcription factors involved in inflammation. **(E)** Expression of a subset of genes within the different groups of intestinal epithelial cell functions described in **(D)**, given as normalized reads. The first 12 panels show genes related to signal transduction, transcription, and translation; the latter show effector genes important for intestinal epithelial cell homeostasis. See main text for details. All displayed genes in the figure panels were differentially regulated with an adjusted P-value <0.05.

To examine how general functions in differentiated colonoids are directly affected by physiological hypoxia, we analyzed an in-house bulk RNA-seq dataset comparing fully differentiated, untreated colonoids cultured continuously at 20% oxygen with colonoids cultured at 2% oxygen for the last 40 hours [(38] and GSE172404). Reduction in oxygen level from 20% to 2% gave 2384 differentially expressed protein-coding genes, of which 1192 genes were downregulated and 1192 genes were upregulated (adjusted P-value <0.05). PCA of the complete dataset demonstrates that 9% (PC2) and 6.3% (PC3) of the transcriptional variation separated the two levels of oxygenation into distinct clusters of gene expression (**Figure 7A**). Gene enrichment analyses revealed that reduced oxygen concentration induced upregulation of networks related to crucial epithelial functions such as cell adhesion, WNT signaling, and epithelial-to-mesenchymal transition, as well as key hypoxia-related networks such as angiogenesis and blood vessel morphogenesis (**Figure 7B**, upper panel). Among downregulated networks, translation in mitochondria was highly significant (**Figure 7B**, lower panel). When exploring differentially expressed genes within these networks further (**Figures 7C-E**), we found regulation of 119 of the 369 hypoxia-related genes (**SF1** File, sheet 14), with 89 upregulated and 30 downregulated in 2% oxygen compared to 20% oxygen (**Figure 7C**, **SF1**, sheet 14).

The differentially expressed genes (**Figure 7**) were involved in all levels of cellular signaling networks, such as receptors and protein-tyrosine kinases (*IL10RB*, *IL22RA*, *IFNAR2, NOD2, CXCR4, IFNGR2*, *JAK1, JAK3*), transcription factors (*FOXO3*, *FOSL2, JUN, JUNB, JUND*), histone demethylases (*KDM2A, KDM3A, KDM4B, KDM5B*) and regulators of translation (*NARS*, *WARS*). Thus, a short-term (40 hours) reduction in oxygen level was associated with gene expression and translation alterations that may impact a broad spectrum of cellular responses. Indeed, among effector genes differentially expressed between 20% and 2% oxygen, we found genes related to stemness and differentiation (*EFNA1, EFNA3, EFNA4, PCNA, MYCBP, E2F2*, *E2F3*, *E2F4, KLF4, TFF1, TFF2, TFF3, MUC17, MUC20, MUC20-OT1)*, genes involved in epithelial cell junctions (*TJP-3, CLDN1, CLDN2, CLDN4, GJB3, CEACAM1, CTNNB1*), genes involved in inflammation (*IL1A*, *IL1B*, *TGFB1*, *IL18*) and genes related to antigen presentation through MHC class I (*CIITA*, *HLA-A*, *HLA-B*, *HLA-C*) (**Figures 7D, E**).

Regarding the hypoxia response, we found, in line with previous observations, an increase in classical HIF-1 target genes *CA9* and *VEGF-A* (38). Furthermore, we found an upregulation of genes associated with glycolysis and lactate production (*ALDOC, ENO1, PGK1, SLC2A1, HK1, HK2, LDHA, SLC16A3*). In contrast, mitochondrial genes related to oxidative metabolism were decreased (*NDUFA9, NDUFAF4, NDUFB6, PPARGC1A*). As expected, genes encoding proteins that protect against oxidative stress *(PARP1, NQO1, and TXNRD2*) were affected in 2% oxygen (**Figure 7D**) as oxidative metabolism was reduced, while several genes coding for dual oxidases (*DUOX1, DUOX2, DUOXA1, DUOXA2*) were upregulated in 2% oxygen. Moreover, the apoptosis inducers BAD and BIK were upregulated. However, expression of the BAX gene, coding for apoptosis regulator BCL-2-like protein 4 (53), was downregulated, and several apoptosis inhibitors (*BCL2L1, BNIP3L, BNIP3, and CTNNB1*) were upregulated (**Figure 7D**). These results support our previous observations that short-term reduction in oxygen concentration did not induce cell death (38). Overall, we found that genes related to critical cellular traits in the epithelium were altered in the colonoids when reducing oxygen from 20% to 2%.

## Discussion

This study is the first aiming to evaluate whether IEOs from the human colon can be cultured from single cells to fully differentiated colonoids in a continuously low oxygen environment close to *in vivo* conditions (physioxia). We examined how colonoids grew, differentiated, and responded to extracellular signals at supraphysiological 20% oxygen and an oxygen environment resembling physiological hypoxia with the 2% concentration based on our previous study showing that human differentiated colonoids adapted well to short time low (2%) oxygen (38). Because the oxygen concentration in colon crypts is higher than for differentiated apical cells, our first question was whether stem cells would be able to proliferate and form 3D colonoids at 2% oxygen. Microscopy and image analysis showed that stem cells established and grew at 2% oxygen, often thriving better than at 20% oxygen. We used the linear mixed model (LMM), previously utilized to assess inter-lab growth rate differences in cancer cell lines (54), to asses colonoid growth patterns. LMM allowed us to dissect to what degree different variables affected colonoid growth. We found a significantly reduced cell mass for colonoids cultured in 20% compared to 2% oxygen. The day of image acquirement was the variable having most impact on cell mass, but it also interacted with the oxygen concentration variable. From the establishment until day six, colonoid growth was similar in 2% and 20% oxygen. After day six, some independent experimental replicates had diverging growth curves with largest differences in the exponential growth phase. This indicates that a lower oxygen environment enables stem cells to proliferate faster in the initial lag phase, but a more detailed analysis is needed to establish the direct effects of oxygen on proliferation in stem cells *vs.* differentiated cell types. However, for non-intestinal stem cells, the benefits of a lower oxygen concentration include reduced oxidative stress, increased self-renewal, and proliferation (55, 56). As discussed below, our transcriptomic data from both scRNA-seq and bulk RNA seq supported that physioxia regulates mitotic cell cycle processes in colonoids as well.

To identify cells with proliferation potential, we stained with KI67 which was significantly more expressed in undifferentiated colonoids in both 2% and 20% oxygen. Protein expression of MUC2 and CK20 was significantly higher in differentiated colonoids than in undifferentiated colonoids, with the same tendency for CGA. There were no significant differences in 2% compared to 20% oxygen for any cell marker. Thus, human colonoids can be established from single stem cells which proliferate (KI67) and differentiate into colonoids with goblet cells (MUC2), absorptive (MUC2 negative, CK20 positive), and enteroendocrine (CGA) cells in both 2% and 20% oxygen. Some CK20-positive differentiated cells retained proliferating potential (KI67 positive) at 2% and 20% oxygen. A recent study determined that a Wnt-3A-, R-spondin- and Nogging-free medium is optimal for colonoid differentiation (57). Our differentiation media had less Wnt-3A than the growth medium (5% *vs*. 50%) but was not Wnt-3A free and may have stimulated more growth with colonoids having a higher proportion of cells with proliferation potential. Since the colonic epithelium has a proliferation niche at the crypt bottom (58), it may be beneficial to retain some proliferating cells during colonoid differentiation to resemble *in vivo* characteristics. Further studies are needed to evaluate the effects of media compositions, as well as oxygen gradients within the organoid cultures (59) for various experimental approaches.

To capture transcriptome-wide effects of oxygen at single-cell resolution, we performed a scRNA-seq on colonoids from four different conditions: undifferentiated and differentiated at 2% or 20% oxygen. Others have used scRNA-seq to discover novel cell types within the intestinal epithelium, heterogeneity in colorectal tumors, and functional cell alterations in IBD (52, 60). A supervised dataset based on curated cell marker lists from bulk RNA-seq of undifferentiated and differentiated colonoids cultured at 20% oxygen was used to identify scRNA-seq cell clusters. A substantially lower number of genes were detected by scRNA seq compared to bulk RNA seq, probably due to e.g., technical reasons or dropout events (61). Genes like *LGR5* and *CHGA* appeared only in bulk-RNA analysis, but gene-combinations detected stem-, progenitor, and differentiated cell clusters in both 2% and 20% oxygen. Although most cell marker genes were equally expressed, we did find some oxygen-dependent differences. Undifferentiated colonoids cultivated at 2% oxygen had higher fractions of cells expressing stem cell markers like *ASPM*, *BIRC5*, and *MKI67* than at 20% oxygen. ASPM contributes to spindle organization, spindle positioning, and cytokinesis in dividing cells (62). Martini et al. showed that BIRC5 (also known as Survivin) in the mouse colon is restricted to the crypt stem cells and transit amplifying cells (63), and that BIRC5 is essential for the maintenance of stem- and transit amplifying cells. KI67 is expressed by actively dividing cells, not in the G_0_-phase (64). We also identified a cluster of late progenitors and differentiated cells in undifferentiated colonoids cultivated at 20% oxygen but not at 2% oxygen, suggesting that cell composition in IEOs can be better tuned during physioxia. Enrichment analysis of the stem cells cluster in 2% oxygen showed significantly increased proliferation-related cell cycles. *Cyclin G*, -*G2*, -*A*, and -*A2* were among the upregulated genes, indicating that several critical cell division processes are upregulated in stem cells cultured in 2%. As described above, colonoids cultured in 2% oxygen had an increased growth pattern, suggesting that stem cells at physoxia self-renew and proliferate more frequently than in 20%. Our findings support research into stem cell oxygen environments in other tissues where atmospheric oxygen concentrations can impair stem cell characteristics (65, 66).

Enrichment analysis of genes in the differentiated cell clusters in 2% and 20% oxygen showed that “*Responses to hypoxia*” and “*Responses to reduced oxygen level”* were highly upregulated in 2%, although hypoxia-related genes could be detected in 20% oxygen as well. One of the upregulated HIF-1 targets was *Gal-1*, which has anti-inflammatory effects by modulating innate and adaptive immune cells’ fate and function (67). *Gal-1* is upregulated in active ulcerative colitis, and Crohn’s disease, which the authors hypothesize is due to *Gal-1* aiding in inflammation resolution (68). Treatment with Gal-1 in 2,4,6-trinitrobenzenesulfonic acid-induced colitis models has shown improved inflammation resolution (69). HIF-1α is degraded in the presence of high oxygen while it is stabilized in lower oxygen levels. Physiological hypoxia in the colonic epithelium stabilizes HIF-1, which is important for barrier integrity, xenobiotic clearance, and cellular metabolism (29, 57, 70). Furthermore, HIF-1 is central in the interplay between the microbiome, intestinal epithelial cells, and the immune system (1, 38, 71), and high oxygen degrades HIF-1 inducing dysbiosis. HIF-1 also regulates creatine metabolism, vital for barrier integrity in IECs (72). Barrier-related genes were upregulated in the differentiated cell clusters in both 2% and 20% oxygen, with the Solute Carrier Family 6 Member 8 (*SLC6A8*) higher expressed at 2% oxygen. IECs express *SLC6A8* to facilitate creatin uptake for energy metabolism (73). Barrier proteins constituting tight-junction and adherent junction proteins are energetically demanding because they are stabilized by actin polymerization and myosin activity (73), requiring up to 20% of the IEC’s available energy (74). *SLC6A8* loss in knockout mice resulted in impaired barrier formation (73), and in, ulcerative colitis patients, a disease hallmarked by barrier defects, *SLC6A8* is downregulated compared to in healthy colon (73).

Intestinal epithelium expresses multiple cytokine receptors and pattern recognition receptors (PRRs) providing crosstalk between microbiota, immune cells, and cell types of the epithelial lining (5). PRRs, like toll-like receptors (TLRs), binding conserved microbial components, initiate signaling cascades to induce interleukins, interferons, chemokines, and antimicrobial peptides. Previously, we have shown that colonoids cultured at 20% oxygen associated stronger to inflammation-related gene networks than colonoids cultured at 2% oxygen for the last 40 hours (38). IBD-relevant proinflammatory cytokines TNF/IL17 increased the expression and release of chemokines which attract neutrophil granulocytes and monocytes at both 20% and 2% oxygen (75, 76), but with a reduction in TNF and TNF/IL17 induced responses in 2% oxygen, indicating anti-inflammatory traits of physioxia. In the present study, we treated the colonoids with TNF and the TLR3 ligand Poly(I:C), and analyzed the conditioned medium for chemokines and NGAL, which is expressed in IECs during active Crohn’s colitis, ulcerative colitis, and collagenous colitis (8, 77–79). Thus, NGAL represents a pro-inflammatory activated epithelium. In the colonoids cultured at 20% or 2% oxygen from single cells to differentiation, we found that TNF/Poly(I:C) induced the release of CXCL2, CXCL5, CXCL6, CXCL10, CXCL12, CX3CL1, CCL25, and NGAL at both 20% and 2% oxygen. We found a tendency for decreased pro-inflammatory response to TNF + Poly(I:C) in 2% oxygen, with a significant difference in CXCL10 and CX3CL1 in colonoid medium at 2% *vs*. 20%. Chemokines stimulate circulating leukocytes to migrate toward inflammatory sites (80), and during IBD CX3CL1 CXCL2, -5, -6 are chemoattractants for neutrophils (81). CXCL10 is upregulated during active IBD, attracting activated T-lymphocytes, particularly Th1 cells (75, 82, 83), while CCL25 directs both T-lymphocytes and dendritic cells (81). The results align with our earlier findings after stimulation with TNF/IL17 and, as discussed (38), corresponds to the anti-inflammatory traits of physiological hypoxia seen in other experimental systems (84, 85). Unphysiological high oxygen levels might exaggerate inflammatory responses in the colonic epithelium and Pavlidis et al. recently showed that IBD patients with a heterogenous cytokine response were associated with biological drug resistance (86). Future work should examine if controlling the oxygen environment *in vivo* alters the cytokine response from IECs, which could increase the efficacy of biological therapeutics.

To further examine the direct effects of physoxia *vs* supraphysiological oxygen level, we did an enrichment analysis of our in-house bulk RNA-seq dataset comparing differentiated, untreated colonoids cultured continuously at 20% oxygen with colonoids cultured at 2% oxygen for the last 40 hours (38). Aligning with others, we find that the oxygen level directly affects transcriptional regulation. The transcription factor FOXO3, with multiple roles in cellular stress responses (49), regulates the adaptation to hypoxia by reducing mitochondrial mass and oxygen consumption during HIF-1 activation (50), while AP-1 proteins, including JUN, JUNB, and FOS, are activated by hypoxia and to cooperate with HIF-1 to increase gene expression (51). Reduced oxygen concentration may induce histone modifications, and we found increased expression of histone demethylases (KDMs) (52) when reducing oxygen from 20% to 2% in colonoids. The observed downregulation of the translation factors NARS and WARS in 2% *vs*. 20% oxygen may reflect lower oxygen consumption and downregulation of translation in mitochondria. In addition to the major regulated gene expression networks related to cell differentiation, barrier function, and metabolism; genes associated with cytokine signaling, inflammation, and antigen presentation were differentially expressed between 20% and 2% oxygen. Consistent with our findings, others have shown that hypoxia strengthens tissue integrity affecting barrier function (87), as part of the protective cellular responses triggered by hypoxia. Intriguingly, Matthijsen et al. (88) showed that epithelial lining repair following small intestinal ischemia protected against inflammation. Stem cell proliferation and differentiation, as well as the intestinal trefoil factors (TFF1-3), are involved in the repair process. Overall, we found that in addition to increased classical HIF-1 target genes, short-time reduction of oxygen directly altered genes related to critical cellular traits in human colonoids.

This study aimed to assess whether human 3D colonoids could be cultivated in a 2% oxygen environment. A possible limitation is that the pericellular oxygen environment was not measured. Correlation between the environmental and pericellular oxygen environment is ruled by the physics of gas diffusion and oxygen distribution in cell cultures (89). Factors influencing this include media thickness, media mixing, connective forces, and cellular oxygen consumption. Okkelmann et al. imaged mouse small intestinal organoids in Matrigel, finding homogeneous oxygen distribution but with pericellular oxygen variations, perhaps dependent on inter-organoid variation in oxygen consumption (90). The optimal culturing condition to mimic *in vivo* conditions is an ongoing debate with several studies supporting physiological hypoxia (56). However, in3certain situations, a low oxygen environment could be disadvantageous. Wang et al. identified a Hopx^+^ colitis-associated regenerative stem cell contributing to mucosal repair in mice, showing that regeneration after mucosal injury is impeded in a hypoxic environment (2% oxygen) mediated by endoplasmic reticulum stress (91). In the present study, with human colonoids in a homeostatic environment, physiological hypoxia appeared beneficial for stem cell proliferation. The studies were performed in different species and different situations (homeostasis *vs*. injury repair), complicating comparison. Sabui et al. showed that hypoxia (1% oxygen or chemically induced) inhibited thiamin pyrophosphate and free thiamin uptake in colonic NCM460 cells. Dietary thiamin is primarily absorbed in the proximal part of the small intestine (92), while microbiota-derived thiamin can be absorbed by colonic epithelial cells (93). Contrarily, several studies show that physiological hypoxia is beneficial for colonic epithelium barrier integrity, cell metabolism, nutrient absorption, and maintaining a communalistic microbiome (1, 23, 31).

In summary, refined patient-derived organoid model systems are effective tools for understanding the interplay between oxygen level, microbiota, and innate immune signals in health and disease. IEOs are also valuable models for, e.g., colon cancer (94) and studies of enteric infections (95, 96). However, the relatively hypoxic gut environment influencing barrier function and inflammatory tone is not appropriately modeled by standard atmospheric oxygen culture conditions. Our data further support that colonoid studies can and should be performed in physioxia if physiological resemblance to the *in vivo* conditions is important. Stabilizing HIF-1α in the colonoids mimics HIF-1α stabilization *in vivo*, increasing the colonoid model’s translational value. Culturing colonoids in a low oxygen environment might benefit co-culturing studies with bacteria. In addition, culturing IEOs at various oxygen concentrations can give valuable information about the effects of oxygen on different cell types in health and diseases.

## Data availability statement

The original contributions presented in the study are publicly available. This data can be found here: https://www.ncbi.nlm.nih.gov/geo/- Accession Numbers - GSE218623, GSE217663, GSE172404.

## Ethics statement

The studies involving human participants were reviewed and approved by Central Norway Regional Committee for Medical and Health Research Ethics. The patients/participants provided their written informed consent to participate in this study.

## Author contributions

TB and AKS supervised the study. GAW, SG, IB, HKS, and TB contributed to the experimental design, generated, and analyzed data. AEØ and AKS collected and characterized patient samples. AF performed bioinformatic analysis. GAW, SG, HSK, AF, and TB made figure panels. GAW and TB drafted the manuscript. All authors contributed to the article and approved the submitted version.
