# Supplementary figures and images for: Physiological hypoxia improves growth and functional differentiation of human intestinal epithelial organoids
Source: Front Immunol. 2023 Jan 27;14:1095812. doi: 10.3389/fimmu.2023.1095812 (PMC9922616; doi:10.3389/fimmu.2023.1095812)

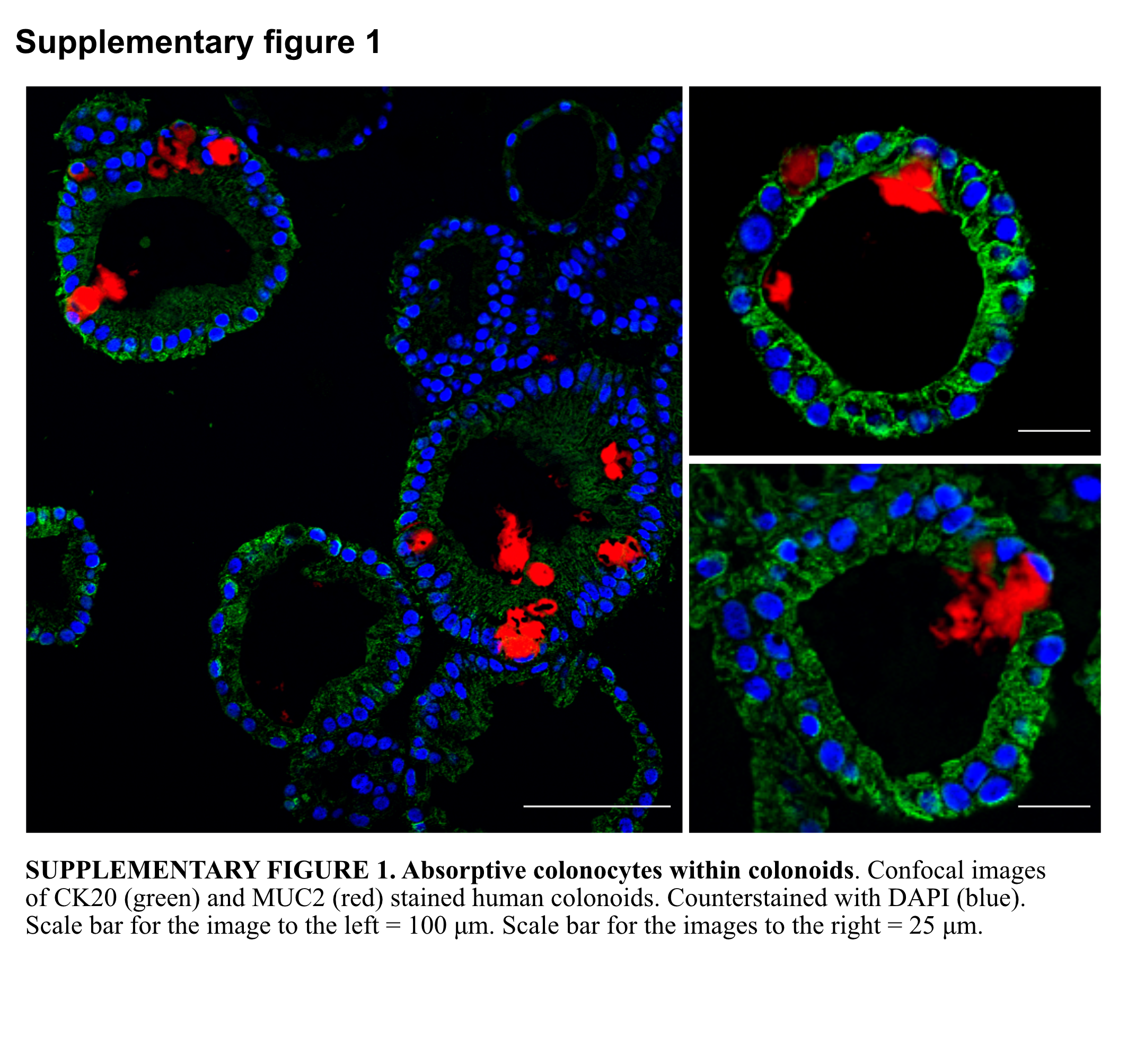

Supplement: Supplementary file 1 [file Image_1.tiff]

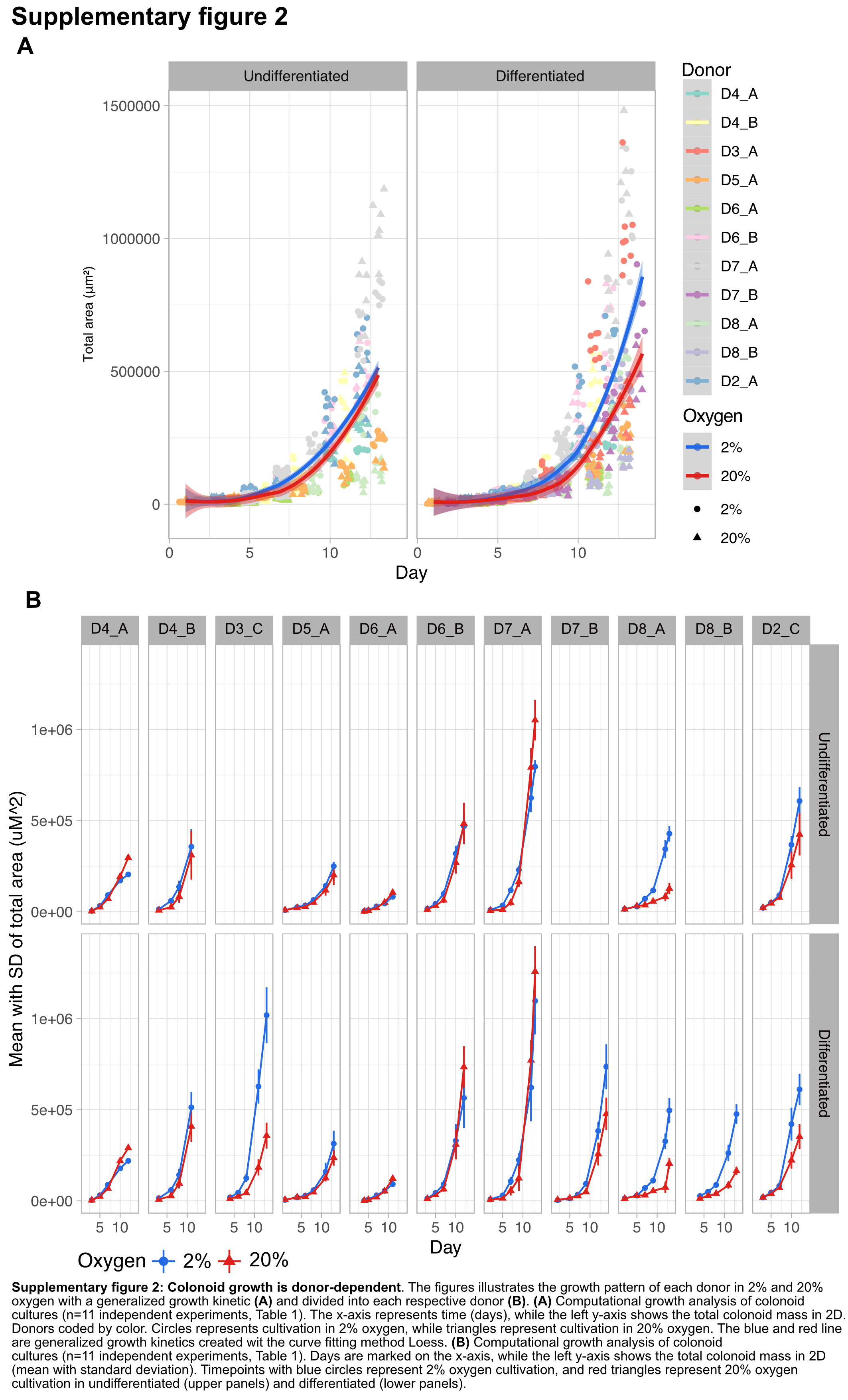

Supplement: Supplementary file 2 [file Image_2.tiff]
